# Rare inborn errors of metabolism with movement disorders: a case study to evaluate the impact upon quality of life and adaptive functioning

**DOI:** 10.1186/s13023-014-0177-6

**Published:** 2014-11-26

**Authors:** Hendriekje Eggink, Anouk Kuiper, Kathryn J Peall, Maria Fiorella Contarino, Annet M Bosch, Bart Post, Deborah A Sival, Marina AJ Tijssen, Tom J de Koning

**Affiliations:** Department of Neurology, University of Groningen, University Medical Center Groningen, Hanzeplein 1, 9700 RB Groningen, The Netherlands; Department of Neurology, University of Amsterdam, Academic Medical Centre, Meibergdreef 9, 1105 AZ Amsterdam, The Netherlands; Department of Neurology, Haga Ziekenhuis Teaching Hospital, Leyweg 275, 2545 CH The Hague, The Netherlands; Department of Paediatrics, University of Amsterdam, Academic Medical Centre, Meibergdreef 9, 1105 AZ Amsterdam, The Netherlands; Department of Neurology, Radboud University Nijmegen Medical Centre, Geert Grooteplein-Zuid 10, 6525 GA Nijmegen, The Netherlands; Department of Genetics, University Groningen, University Medical Center Groningen, Hanzeplein 1, PO Box 30001, 9700 RB Groningen, The Netherlands

**Keywords:** Inborn errors of metabolism, Movement disorders, Quality of life, Adaptive functioning, Dystonia, Myoclonus, Ataxia

## Abstract

**Background:**

Inborn errors of metabolism (IEM) form an important cause of movement disorders in children. The impact of metabolic diseases and concordant movement disorders upon children’s health-related quality of life (HRQOL) and its physical and psychosocial domains of functioning has never been investigated. We therefore conducted a case study on the HRQOL and development of adaptive functioning in children with an IEM and a movement disorder.

**Methods:**

Children with co-existent IEM and movement disorders were recruited from paediatric outpatient clinics. We systematically collected clinical data and videotaped examinations. The movement disorders were diagnosed by a panel of specialists. The Pediatric Quality of Life Inventory 4.0 and the Vineland Adaptive Behavior Scale were used to assess the HRQOL and adaptive functioning, respectively.

**Results:**

We recruited 24 children (10 boys, mean age 7y 5 m). Six types of movement disorders were recognised by the expert panel, most frequently dystonia (16/24), myoclonus (7/24) and ataxia (6/24). Mean HRQOL (49.63, SD 21.78) was significantly lower than for other chronic disorders in childhood (e.g. malignancy, diabetes mellitus, rheumatic disease, psychiatric disorders; *p* <0.001) and tended to diminish with the severity of the movement disorder. The majority of participants had delayed adaptive functioning, most evident in their activities of daily living (51.92%, SD 27.34). Delay in adaptive functioning had a significant impact upon HRQOL (*p* = 0.018).

**Conclusions:**

A broad spectrum of movement disorders was seen in patients with IEM, although only five were receiving treatment. The overall HRQOL in this population is significantly reduced. Delay in adaptive functioning, most frequently seen in relation to activities of daily living, and the severity of the movement disorder contribute to this lower HRQOL. We plead for a greater awareness of movement disorders and that specialists should be asked to diagnose and treat these wherever possible.

**Electronic supplementary material:**

The online version of this article (doi:10.1186/s13023-014-0177-6) contains supplementary material, which is available to authorized users.

## Background

Inborn errors of metabolism (IEM) form a heterogeneous group of rare inherited disorders in which the synthesis, metabolism, transport and/or storage of metabolites or molecules is disturbed. These changes can affect all organs and result in a variety of symptoms. The central nervous system is frequently involved, with disorders often manifesting as epilepsy and psychomotor retardation. Movement disorders, although less frequently documented, may also be present [1,2].

IEM contribute to a significant proportion of childhood movement disorders, classified as ataxia, hypokinetic or hyperkinetic. The last category is further subdivided into dystonia, myoclonus, chorea, ballism, tremor and tics. An accurate classification is important for diagnosis, ongoing management and treatment choices. However, there is little data on which movement disorders are most prevalent in IEM. In addition, identifying movement disorder subtypes, especially in children, can be challenging as these are often associated with secondary structural lesions. These may also precipitate additional neurological features e.g. spasticity, epilepsy, psychomotor retardation or other movement disorders, resulting in a complex clinical picture [3].

As with other chronic, disabling conditions, IEM are likely to impair physical functioning and social participation. As a result, assessing health-related quality of life (HRQOL) has emerged as an important outcome measure in chronic illnesses of childhood [4,5]. HRQOL comprises the subjective perception of the impact of a chronic illness on physical, psychological, social and occupational functioning and its use has been established in a broad range of childhood conditions [6,7].

To date little is known about how much impact IEM have on children’s HRQOL. Studies in adequately treated phenylketonuria (PKU) report a similar HRQOL to control populations [8,9]. However, PKU is detected by screening in the newborn period thereby allowing early therapeutic intervention and minimizing neurological damage. This population may therefore not be representative of all IEM as a significant number of IEM are detected when neurological impairment has already occurred. For example, recent papers on galactosaemia and Hunter syndrome (mucopolysaccharidosis type II) revealed a markedly impaired HRQOL in the physical and psychosocial domains of functioning [10,11]. To our knowledge, no previous studies have investigated the impact of a broader range of IEM with secondary movement disorders upon quality of life of these patients.

IEM in children are frequently associated with developmental delay in one or more domains of functioning [12]. The level of disability may vary considerably between patients, suggesting that this is an important factor when assessing HRQOL. Consequently, adaptive functioning, defined as ‘the collection of conceptual, social and practical skills that have been learned by children in order to function in their everyday lives’, has become an important outcome measure [13]. Our literature search found no reports combining both HRQOL and adaptive functioning tools in assessing patients with IEM.

We systematically assessed the types of movement disorders observed in a cohort of patients with an established IEM diagnosis. We are the first to use systematic and standardised questionnaires to assess HRQOL and adaptive functioning, thereby highlighting areas of impairment in the context of these disorders.

## Methods

### Participants

Participants younger than 19 years of age were recruited between January and July 2007 from two Dutch university medical centres (Wilhelmina Children’s Hospital, Utrecht and Academic Medical Centre, Amsterdam). All patients with a confirmed IEM and a suspected movement disorder were asked to participate. After enrolment, informed consent or third party assent was obtained in all cases. The study was approved by the medical research and ethics committees of both centres.

### Data collection

All participants were assessed in their home environment and a videotaped examination was performed using a standard protocol. Past medical history relating to the diagnosis of the metabolic disorder, and past and present medical therapies and their efficacy were collected from the clinical notes. Three movement disorder specialists (MFC, BP, MT) were blinded to the clinical diagnosis and asked to classify the movement disorder and its severity according to a 5-point Likert scale based on the videotaped examination. Severity scores ranged from 1 (minimal), 2 (mild), 3 (moderate), 4 (severe) to 5 (very severe).

The Dutch Generic Core Scale of the Pediatric Quality of Life Inventory (PedsQL) 4.0 and the Vineland Adaptive Behavior Scale (VABS) questionnaires were completed by the parents in each case [14]. The PedsQL was used to determine the HRQOL. This questionnaire asks parents to indicate to what extent the child had a problem with each item the past month. Items are subdivided in physical (8 items; walking, running, exercise, lifting, self-care, pain, tiredness), emotional (5 items; anxiety, sadness, angriness, trouble sleeping, worrying), social (5 items; getting along, friends, teasing, keeping up with others, playing) and school-related domains (5 items; attention, forgetting, schoolwork, missing school because of sickness or doctor visits). Scores were calculated by the sum of all scores divided by the number of answered items. Results were compared to parent-proxy reports of the PedsQL for other chronic stigmatizing conditions [7,15-18].

The VABS, validated in the Netherlands for children with or without cognitive impairment, was used to determine age-appropriate adaptive functioning in areas of communication, daily living skills and socialisation [19]. The total score and scores of the three sub-domains were converted to an estimated age of development using validated age-equivalents. Percentage of development was calculated by dividing estimated age of development by biological age. Percentage of delay was calculated by subtracting the percentage of development from 100%.

### Statistical analysis

Statistical analysis included Mann Whitney U and t-tests, as well as linear regression analysis where appropriate. Results were corrected for multiple comparisons using the Bonferroni-Holm correction.

## Results

A total of 24 children were recruited (10 boys, 14 girls, mean age 7y 5 m). This cohort contained 12 different IEM diagnoses, with organic acidurias (11/24) and respiratory chain defects (5/24) being the most common. Table 1 presents the demographic characteristics and IEM diagnoses. MRI data were available in 17 patients, showing abnormalities in twelve. Most common white matter abnormalities (5/17), atrophy (4/17) and basal ganglia abnormalities (4/17) (Additional file 1: Table S1).

**Table 1:** **Demographics and details of IEM diagnosis (** ***n*** **= 24)**

	**No. of cases**
Sex	
Male	10
Female	14
Mean age (years, months) (SD)	7y 5 m (4y 1 m)
Metabolic diagnosis	
Respiratory chain defects	5
Organic acidurias	10
Glutaric aciduria type I	4/10
Propionic acaduria	2/10
Homocystinuria	2/10
Maple syrup urine disease	1/10
Methylmalonic aciduria	1/10
Disorders of carbohydrate metabolism	1
Galactosaemia	1/1
Neurotransmitter defects	2
PTPS deficiency	1/2
AADC deficiency	1/2
Other	6
CDG 1a	3/6
AASA deficiency	1/6
MCT-8 deficiency	1/6
Nonketotic hyperglycinemia	1/6

*IEM* Inborn error of metabolism, *AADC* Aromatic amino acid decarboxylase, *AASA* Alpha-aminoadipic semialdehyde, *CDG* Congenital disorder of glycosylation, *MCT-8* Monocarboxylate transporter 8, *PTPS* 6-pyruvoyl-tetrahydropterin synthase, *SD* Standard deviation.

Based on videotape evaluation by the panel of movement disorder specialists, dystonia was the most frequently observed movement disorder (16/24, 10 generalised, 4 segmental and 2 focal), followed by myoclonus (7/24) and ataxia (6/24). In over one-third of cases (9/24), two or more movement disorders were observed simultaneously (Additional file 1: Table S1).

**Figure 1:**
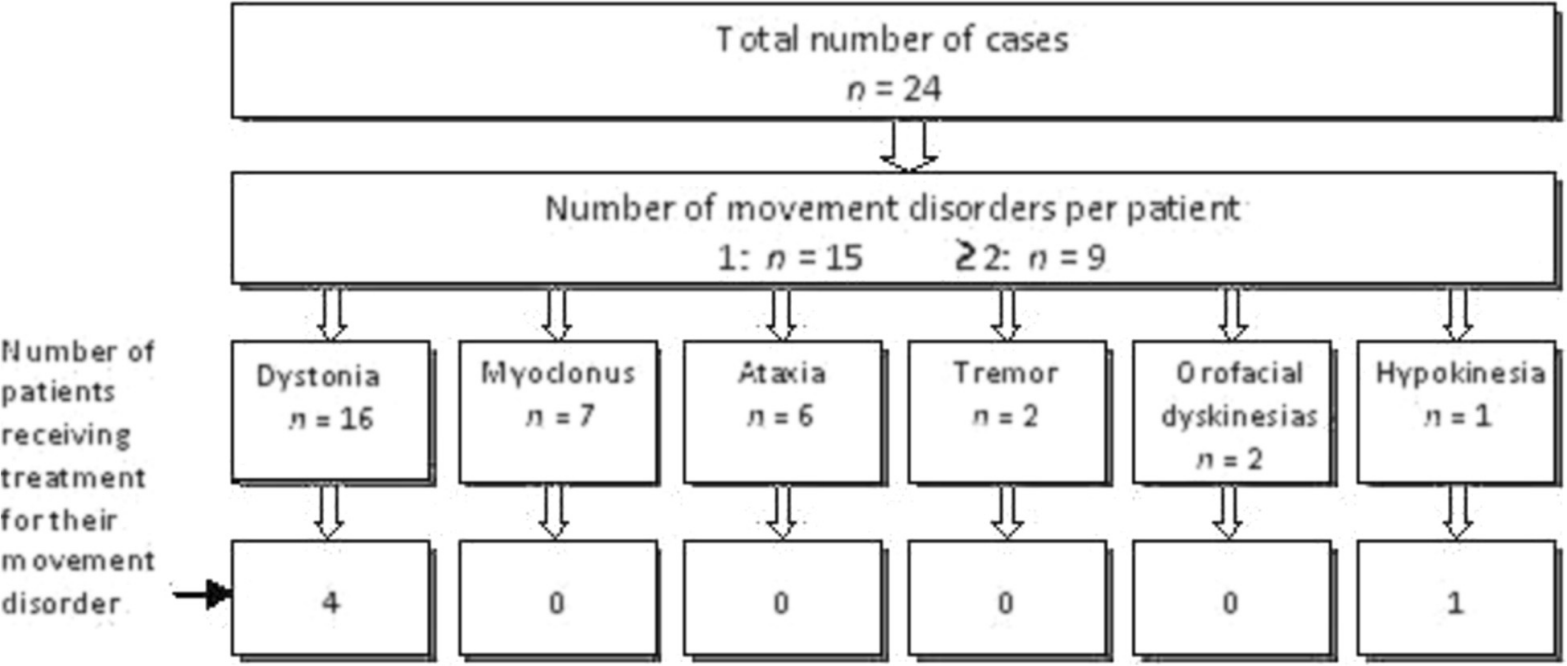
**Diagrammatic representation of the number, type and treatment of movement disorders.** Scheme showing the number of movement disorders per patient, and the number of patients for each movement disorder subtype. The bottom row of boxes indicates the number of cases receiving treatment in each subgroup.

Disease specific therapy was provided in the majority of the patients, mostly consisting of dietary measures (Additional file 1: Table S1). Medical therapy for treatment of the movement disorders had been prescribed in only five cases (Figure 1): in four patients, dystonia was the predominant movement disorder, while the fifth patient had hypokinesia with more subtle dystonia. Two dystonic patients with Glutaric Aciduria (GA) type 1 received trihexyphenidyl and one Methylmalonic Aciduria patient with dystonia and orofacial dyskineias was treated with trihexyphenidyl combined with clonazepam. In all three cases, the parents reported a beneficial effect, particularly in reducing the mobile component of dystonia. The fourth dystonic patient, who had 6-pyruvoyl-tetrahydropterin synthase (PTPS) deficiency, received levodopa-carbidopa both to restore normal neurotransmission of dopamine as well as minimise dystonia symptoms. Parents reported a marked response as patient showed only a mild dystonia of the limbs during walking with this treatment strategy. The patient with a predominant hypokinetic movement disorder had an underlying diagnosis of Aromatic Amino Acid Decarboxylase (AADC) deficiency. She had previously been treated with pramipexol and tranylcypromine, but stopped the latter due to adverse side effects.

### Health-related quality of life

PedsQL scores were found to be independent of age and sex. When compared to control data, patients in this study had a statistically significant lower HRQOL score in each functional sub-domain as well as in the total score (Table 2) [14].

**Table 2:** **HRQOL scores of children with IEM and movement disorders compared to scores in healthy children**

	**IEM and movement disorders** ***n*** **= 24**	**Healthy Dutch population** ***n = *** **87**	**t-value**	**CI 95%**	***p*** -value*
	**Mean value (SD)**	**Mean value (SD)**			
Total score	49.63 (21.78)	87.60 (11.00)	-8.21	-28.78 to -47.16	**<0.001**
Physical functioning	39.71 (24.56)	92.20 (9.10)	-10.44	-43.31 to -63.67	**<0.001**
Emotional functioning	63.50 (23.18)	81.10 (17.40)	-3.42	-7.38 to -27.82	**<0.001**
Social functioning	49.71 (29.18)	90.30 (14.00)	-6.57	-25.32 to -52.86	**<0.001**
School functioning	51.29 (32.05)	82.50 (16.30)	-4.49	-17.41 to -45.01	**<0.001**

HRQOL scores were measured on the PedsQL 4.0 Generic Core Scales. Parent proxy-reports of children with IEM and movement disorders were compared to scores of healthy Dutch children [14].

*CI* Confidence interval, *IEM* Inborn error of metabolism, *SD* Standard deviation, *significance level after Bonferroni-Holm correction for multiple comparisons.

Bold text: statistically significant.

When compared to published data for children with other chronic, disabling disorders, this cohort was found to have a significantly lower total HRQOL: malignancy (mean 68.47, SD 19.22, t(583) = -4.17, *p* < 0,001), diabetes mellitus (mean 76.62, SD 14.08, t(329) = -5.97, *p* < 0,001), juvenile rheumatic disease (mean 68.73, SD 19.32, t(379) = -4,19, *p* <0,001) and psychiatric disorders (mean 66.90, SD 14.00, t(329) = -3.28, *p* < 0.001) [7,15-17]. There was no significant difference with data for children with cerebral palsy (mean 51.28, SD 18.00, t(246) = -0.36, *p* = 0.721) [18].

Severity scores were available in all but one case. We divided this study cohort based on movement disorder severity (mild to moderate versus marked to severe) and found a significant difference in the physical functioning domain and total HRQOL score; those with a less severe movement disorder reported higher quality of life scores (Table 3).

**Table 3:** **Comparison of HRQOL scores between patients with a less severe and more severe movement disorder**

**Severity of movement disorder**	**Minimal to moderate** ***n = *** **14**	**Marked to severe** ***n*** **= 9**	**U-value**	**Z-value**	***p*** -value*
	**Mean value (SD)**	**Mean value (SD)**			
Total score	59.00 (17.92)	33.67 (19.55)	22.00	-2.60	**0.009**
Physical functioning	49.64 (22.28)	20.44 (18.08)	18.00	-2.84	**0.003**
Emotional functioning	69.57 (20.33)	54.44 (26.75)	40.00	-1.45	0.159
Social functioning	59.86 (26.24)	31.67 (27.04)	48.00	-2.21	0.028
School functioning	57.36 (29.27)	39.22 (35.52)	45.50	-1.11	0.277

HRQOL scores were measured on the PedsQL 4.0 Generic Core Scales. The total cohort was divided in two groups based on the severity of their movement disorder, determined by the expert panel.

*HRQOL* Health-related quality of life, *SD* Standard deviation; *significance level after Bonferroni-Holm correction for multiple comparisons.

Bold text: statistically significant.

### Adaptive functioning

The mean overall delay reported for adaptive functioning was 51.92% (SD 27.34), suggesting a developmental age half that of their chronological age. Delay was seen across all three sub-domains, with the highest rates observed in relation to daily living skills (mean 57.70%, SD 23.80), followed by socialisation (mean 40.92%, SD 33.50) and communication (mean 38.32%, SD 38.39). The extent of delay varied widely among the patients (range 0-90.95%) and was unrelated to age or sex. Adaptive functioning (total VABS score) was found to have a significant impact upon HRQOL using linear regression analysis (F(1,22) = 7.171, *p* = 0.014), accounting for 24.6% of the variance in reported HRQOL (R^2^ = 0.246).

After the Bonferroni-Holm correction, a more severe movement disorder did lead to a significantly larger relative delay in daily living skills (49.00% SD 23.88 vs 71.11% SD 19.15, U(23) = 102.50, Z = 2.489, *p* = 0.011). We saw trends, but no significant differences, with communication (23.92% SD 36.44 vs 60.03 SD 34.37, U(23) = 95.00, Z = 2.018, *p* = 0.046), socialisation (31.74% SD 30.93 vs 56.23 SD 35.35, U(23) = 92.50, Z = 1.859, *p* = 0.062) and total adaptive functioning (43.19% SD 24.46 vs 65.72 SD 28.80, U(23) = 96.00, Z =20.79, *p* = 0.039).

## Discussion

We systematically assessed the types of movement disorder observed in a paediatric IEM population with a broad range of underlying diagnoses. This is the first study to use systematic and standardised tools for assessing HRQOL and adaptive functioning in such a population. We also analysed the influence of movement disorder severity and how delay could impact upon overall quality of life.

Of the domains assessing HRQOL, difficulties with physical functioning were the most frequently reported and formed the largest contributor to reduced quality of life. Overall HRQOL in this population was markedly lower than that of other chronic disabling disorders, e.g. joint disease and diabetes, but similar to that reported in children with cerebral palsy. A previous study analysing HRQOL scores across a range of conditions found those with cerebral palsy to be in the group with the most impaired scores [7]. Both IEM and cerebral palsy are associated with motor impairment and psychomotor retardation, suggesting that these are likely to be important determinants in overall well-being in childhood [12,20]. These results also suggest that while the impact of cerebral palsy upon quality of life is widely acknowledged, the effect of IEM with movement disorders is similar and deserves wider recognition in clinical practice.

Adaptive functioning scores varied widely, with the majority of participants demonstrating delay in at least one domain. This likely reflects the global impact of IEM upon cerebral functioning, rather than damage to specific areas. The extent of the delay also varied widely across all three domains, consistent with the large variation in cerebral damage reported in IEM [1]. Four patients had better communication or socialisation skills than expected for their age. However, these same patients were reported to have significant delays in activities of daily living, resulting in a large overall delay in adaptive functioning. These findings demonstrate the importance of being able to perform basic tasks upon developmental delay.

Overall, adaptive functioning and HRQOL appear to be related; a larger delay was associated with a lower HRQOL. The overall level of adaptive functioning accounted for a significant proportion of the variance in HRQOL and may also partly account for the difference in HRQOL between this cohort and previous studies of PKU patients [8,9]. However, it also demonstrates that in disorders where developmental delay is observed, estimation and allowance for adaptive functioning are important factors in clinical management.

A broad spectrum of predominantly hyperkinetic movement disorders were seen in this cohort. Not all patients showed an anatomical defect on MRI, a relatively small number showed basal ganglia changes. In previous literature, it has been acknowledged that the movement disorders in IEM are often associated with basal ganglia dysfunction. This is however not necessary observed anatomically, for example in neurotransmitter disorders [21]. Of the four patients in our cohort who showed basal ganglia abnormalities on MRI, three suffered from an organic aciduria. Interestingly, all four presented with dystonia as the predominant movement disorder.

The limited number of publications addressing IEM and movement disorders, coupled with the small number of cases in this cohort that had received treatment targeting their movement disorder, suggests there may be poor recognition of movement disorders in IEM populations. Nonetheless, our results show that a more severe movement disorder may be associated with a lower overall quality of life and a larger developmental delay in daily living skills. These results underscore the importance of recognizing movement disorders in complex childhood disorders like IEM.

An accurate diagnosis of a movement disorder is also essential in the choice of therapeutic management and likely efficacy of treatment. This factor was highlighted in our cohort, with only five patients receiving treatment for their movement disorder. Three of these were treated with trihexyphenidyl and one with levodopa/carbidopa, with a good symptomatic response reported in all cases. The fifth was treated with pramipexol for her hypokinesia (frequently used in patients with AADC deficiency). However, the specialists who analysed this patient’s video felt that dystonia was also present, for which she received no specific therapy.

There are several therapeutic options available to treat movement disorders. Besides disease-specific therapy often additional symptomatic therapy is necessary. Examples of symptomatic therapies are trihexyphenidyl or botulinum toxin for dystonia and clonazepam for myoclonus. Especially with the existence of these options and development of novel symptom-specific treatments , for instance like deep brain stimulation for childhood dystonia, an accurate clinical diagnosis by a specialist is becoming increasingly important.

In our study, only patients with a suspected movement disorder were asked to participate. Therefore it is not possible to draw conclusions on the incidence of movement disorders in this population. Also, the studied group is heterogeneous and comprises patients with diverse and severe disorders. In these patients the presence of movement disorders is just one of the factors affecting the HRQOL. The small sample sizes prevented subgroup analysis to differentiate the impact of type and severity of the movement disorder from the wider complications of the IEM upon HRQOL and adaptive functioning. A larger, multi-centre study, comprising patients with and without movement disorders, would be required to investigate this further.

Furthermore, in this study we collected standardised parental observations rather than self-reported assessments by the children. Although self-reported data is widely acknowledged to be preferential to proxy-reported data, most participants (19/24) in this study were unable to complete the questionnaires themselves. Previous studies found that when children may have communication or cognitive deficits, parental reporting provides a useful alternative tool [22].

## Conclusions

This study demonstrates that HRQOL in children with IEM and co-existent movement disorder is significantly reduced compared to other chronic, stigmatizing disorders. Delay in adaptive functioning and a more severe movement disorder were associated with a lower HRQOL. Accurate classification of movement disorders, particularly in the context of complicated, multi-organ disorders, is likely to aid therapeutic management, especially since effective therapeutic options are available. We hope a greater awareness of the presence of movement disorders and targeted therapies might lead to improved motor function and possibly subsequent overall quality of life.

## Additional file

Additional file 1: Table S1. Patient characteristics, IEM diagnosis, movement disorder diagnosis, MRI abnormalities and treatment strategies (*n* = 24). IEM: inborn error of metabolism, AADC: aromatic amino acid decarboxylase, AASA: alpha-aminoadipic semialdehyde, CDG: congenital disorder of glycosylation, GA: glutaric aciduria; MCT-8: Monocarboxylate transporter 8, PTPS: 6-pyruvoyl-tetrahydropterin synthase. The classification of the intellectual disability is according to the American association on Intellectual and Developmental Disabilities (2010).

## Abbreviations

AADC: Aromatic amino acid decarboxylase
AASA: Alpha-aminoadipic semialdehyde
CDG: Congenital disorder of glycosylation
CI: Confidence interval
HRQOL: Health-related quality of life
IEM: Inborn errors of metabolism
MCT-8: Monocarboxylate transporter 8
PedsQL: Pediatric quality of life inventory
PKU: Phenylketonuria
PTPS: 6-Pyruvoyl-tetrahydropterin synthase
SD: Standard deviation
VABS: Vineland adaptive behavior scale
