# Supplementary material for: Rare inborn errors of metabolism with movement disorders: a case study to evaluate the impact upon quality of life and adaptive functioning
Source: Orphanet J Rare Dis. 2014 Nov 26;9:177. doi: 10.1186/s13023-014-0177-6 (PMC4254263; doi:10.1186/s13023-014-0177-6)
Supplement: Additional file 1: Table S1. — Patient characteristics, IEM diagnosis, movement disorder diagnosis, MRI abnormalities and treatment strategies (n = 24). IEM: inborn error of metabolism, AADC: aromatic amino acid decarboxylase, AASA: alpha-aminoadipic semialdehyde, CDG: congenital disorder of glycosylation, GA: glutaric aciduria; MCT-8: Monocarboxylate transporter 8, PTPS: 6-pyruvoyl-tetrahydropterin synthase. The classification of the intellectual disability is according to the American association on Intellectual and Developmental Disabilities (2010). [file 13023_2014_177_MOESM1_ESM.docx]

**Additional file 1: Table S1: Patient characteristics, IEM diagnosis, movement disorder diagnosis, MRI abnormalities and treatment strategies (*n* = 24)**

| Case | Age/  Sex | IEM | Movement disorder(s) | Intellectual disability | MRI abnormalities | Disease specific treatment | Movement disorder treatment |
| --- | --- | --- | --- | --- | --- | --- | --- |
| 1 | 3/F | AADC-deficiency | Hypokinesia,  Focal dystonia, Orofacial dyskinesias | Profound | No MRI available | Dietary | Pramipexol, Tranylcypromine |
| 2 | 6/F | AASA-deficiency | Ataxia | Mild | No abnormalities | Dietary |  |
| 3 | 3/M | CDG 1a | Ataxia | Mild | No MRI available |  |  |
| 4 | 6/F | CDG 1a | Generalised dystonia, Ataxia | Profound | Severe global atrophy, signal abnormalities in putamen and globus pallidus |  |  |
| 5 | 7/M | CDG 1a | Ataxia,  Generalised dystonia | Moderate | No MRI available |  |  |
| 6 | 4/M | GA type 1 | Generalised dystonia, Myoclonus | Mild | Arachnoidal cysts anterior to temporal lobe, open Sylvian fissures, increased signal intensity bilateral putamen | Dietary |  |
| 7 | 4/M | GA type 1 | Generalised dystonia, Ataxia | Moderate | Subdural hygromas, Periventricular white matter abnormalities | Dietary |  |
| 8 | 13/M | GA type 1 | Generalised dystonia | Moderate | No MRI available | Dietary | Trihexyphenidyl |
| 9 | 7/F | GA type 1 | Generalised dystonia | Profound | No MRI available | Dietary | Trihexyphenidyl |
| 10 | 10/F | Galactosemia | Focal dystonia, tremor | No | Nonspecific white matter abnormalities, delay in myelination | Dietary |  |
| 11 | 16/F | Homocystinuria | Myoclonus | Moderate | No abnormalities | Dietary |  |
| 12 | 18/F | Hymocystinuria  (vit B6 responsive) | Tremor | No | No MRI available | Dietary |  |
| 13 | 3/M | MCT-8 deficiency | Generalised dystonia | Severe | Periventricular white matter abnormalities | Dietary |  |
| 14 | 7/F | Methylmalonic aciduria | Segmental dystonia, Orofacial dyskinesias | Profound | Bilateral cystic tissue defect of globus pallidus | Dietary | Trihexyphenidyl , Clonazepam |
| 15 | 5/M | MSUD | Segmental dystonia, Myoclonus | Profound | Abnormal signal intensity basal ganglia, capsula interna, pons and medulla oblongata | Dietary |  |
| 16 | 2/M | Nonketotic hyperglycinemia | Ataxia | Moderate | No abnormalities | Dextromethorphan, Sodium benzoate, Folinic acid |  |
| 17 | 6/F | Propionic acidemia | Segmental dystonia | Moderate | No abnormalities | Dietary, Metronidazole |  |
| 18 | 11/F | Propionic acidemia | Myoclonus | Severe | Global signs of atrophy | Dietary, Metronidazole |  |
| 19 | 8/M | PTPS-deficiency | Segmental dystonia | Moderate | Periventricular white matter abnormalities | BH-4,  5-OH-tryptophan, Levodopa-carbidopa |  |
| 20 | 3/F | Respiratory chain defect | Myoclonus | Severe | Enlarged right lateral ventricle | Dietary |  |
| 21 | 5/F | Respiratory chain defect | Generalised dystonia, Myoclonus | Profound | Enlarged ventricles | Dietary |  |
| 22 | 6/M | Respiratory chain defect | Myoclonus | Mild | No MRI available | Dietary,  Co- enzyme Q10 |  |
| 23 | 8/F | Respiratory chain defect | Generalised dystonia | Profound | No abnormalities | Dietary |  |
| 24 | 10/F | Respiratory chain defect | Generalised dystonia | Profound | Periventricular white matter abnormalities, especially occipital | Dietary |  |

IEM: inborn error of metabolism, AADC: aromatic amino acid decarboxylase, AASA: alpha-aminoadipic semialdehyde, CDG: congenital disorder of glycosylation,

GA: glutaric aciduria; MCT-8: Monocarboxylate transporter 8, PTPS: 6-pyruvoyl-tetrahydropterin synthase.

The classification of the intellectual disability is according to the American association on Intellectual and Developmental Disabilities (2010)
